# Enhanced Recovery After Surgery for Pediatric Lung Resection: Effects of a New Protocol

**DOI:** 10.3390/children12121658

**Published:** 2025-12-07

**Authors:** Andrew J. Behrmann, Elizabeth A. Shumway, Brooklyn Campbell, Cannon Dew, Tara Kempker, Jessica Peuterbaugh, Venkataraman Ramachandran, Yousef El-Gohary, Ahmed I. Marwan

**Affiliations:** Division of Pediatric General, Thoracic and Fetal Surgery, Department of Surgery, University of Missouri School of Medicine, Columbia, MO 65212, USA; ajbgk6@umsystem.edu (A.J.B.); easkf9@health.missouri.edu (E.A.S.); bocvgd@health.missouri.edu (B.C.); ccddp9@health.missouri.edu (C.D.); tkempker@health.missouri.edu (T.K.); peuterbaughj@missouri.edu (J.P.); ramachandranv@health.missouri.edu (V.R.); yousef.gohary@health.missouri.edu (Y.E.-G.)

**Keywords:** congenital pulmonary airway malformation, congenital lung lesions, lobectomy, enhanced recovery after surgery

## Abstract

**Background**: Prenatal detection of congenital lung lesions has increased with improved imaging. These abnormalities are safely treated with thoracoscopic lobectomy. We implemented an enhanced recovery after surgery (ERAS) protocol to standardize care and aim to evaluate its safety and efficacy compared to a non-ERAS cohort. **Methods**: A single-center retrospective chart review was conducted for twenty patients (n = 10 ERAS, n = 10 non-ERAS) undergoing thoracoscopic lobectomy from 2014–2024. **Results**: ERAS patients were generally younger at the time of surgery (ERAS: 4.25 ± 2.76 months vs. non-ERAS: 6.45 ± 6.78 months, *p* = 0.17). Postoperative length of stay was shorter in ERAS (1.77 ± 0.60 days) vs. non-ERAS patients (5.25 ± 3.79 days, *p* = 0.03) as well as chest tube duration (ERAS: 1.44 ± 0.73 days vs. non-ERAS 3.64 ± 2.38 days, *p* = 0.01). ERAS patients received lower amounts of opioid analgesics compared to non-ERAS (*p* = 0.0046). Use of the ERAS protocol also decreased cost for the healthcare system compared to non-ERAS patients (*p* = 0.0037). ERAS patients had no reintubations or prolonged air leaks (defined as >48 h), compared to four reintubations (*p* = 0.04) and three prolonged air leaks (*p* = 0.07) in the non-ERAS group. Crucially, there were no complications in the ERAS group, whereas five non-ERAS patients experienced Clavien–Dindo level III (one IIIa, two IIIb, two IVa) complications (*p* = 0.02). **Conclusions**: Our preliminary findings demonstrate the successful integration of a novel ERAS protocol in pediatric thoracoscopic lobectomies and its efficacy in reducing standard post-operative recovery times without an increased rate of complications. Earlier discharge in the ERAS group constitutes less healthcare burden with improved resource utilization and less family, work, and social disruption.

## 1. Introduction

Congenital cystic lung lesions are developmental abnormalities of branching morphogenesis affecting the fetal airway and pulmonary parenchyma. There has been an increase in the diagnosis of CPAMs since the mid-2000, given the advancements and widespread adoption of prenatal imaging [1]. Congenital pulmonary airway malformations (CPAMs) are the most common prenatally diagnosed congenital lung lesions. They are often cystic, non-functional parenchymal tissues that constitute a space-occupying lesion, are associated with an increased risk of infection, neoplasm, and late surgical complications if they are not resected early in life [2,3]. In the United States, it is common practice to surgically resect CPAMs. This has been done with promising clinical results, especially when performed thoracoscopically, due to the pediatric lung’s capacity to undergo post-lobectomy compensatory lung growth and lower complication rates of thoracoscopic surgery [4,5]. However, in other countries such as Canada and various areas in Europe, CPAM management is heterogeneous with some only resected when the patient becomes symptomatic without consensus on surgical approach (e.g., lobectomy versus segmental resection) [6,7]. This difference in management strategy has also naturally led to a difference in the timing of these procedures, with most of them happening at just a few months of age in the United States versus later in life in other countries [3,8].

Enhanced recovery after surgery (ERAS) protocols aim to improve overall outcomes for surgical patients while minimizing their hospital stay by optimizing preoperative status, providing multimodal analgesic regimens with decreased opioid use, and emphasizing early extubation, mobilization, and nutritional support [9,10,11,12]. ERAS protocols also create a standardized pre- and post-operative treatment plan for patients and their care team to follow, which can help reduce the heterogeneity of clinical management within a single institution.

Our institution has a history of variable management strategies and outcomes for congenital lung lesions, even after modernizing our practice with thoracoscopic approaches. The aim of this study is to develop and implement an ERAS protocol for congenital lung lesion resections to standardize our clinical management and decrease the time to discharge for patients in addition to conducting a preliminary comparison of our outcomes before and after protocol implementation. We hypothesize that the implementation of our ERAS protocol will be safe, will not be associated with an increase in complication rates, and will lead to a reduced length of stay (LOS).

## 2. Materials and Methods

For data acquisition, a retrospective cohort analysis was performed on all pediatric lung resections performed for congenital lung lesions at the University of Missouri over a 10-year period. Our protocol was implemented in January 2022. There were 10 non-ERAS patients and 10 ERAS patients. All continuous variables are described as mean ± standard error of the mean. Effect sizes are reported as Cohen’s d for continuous variables and Phi coefficients for categorical variables. We have noticed a significant increase in the number of these patients following the development of our Fetal Intervention Center. The demographics of our patient population are presented in Table 1. One patient in the non-ERAS cohort presented with symptoms of congenital hyperinflation syndrome which required an open resection. All other patients in both ERAS and non-ERAS groups were asymptomatic. All congenital lung lesions in both the pre-ERAS and ERAS cohorts were diagnosed prenatally and followed by maternal–fetal–medicine specialists. Prenatal identification was made via ultrasound either through referral to our maternal–fetal–medicine clinic or through our institution’s fetal care program.

**Figure 1:**
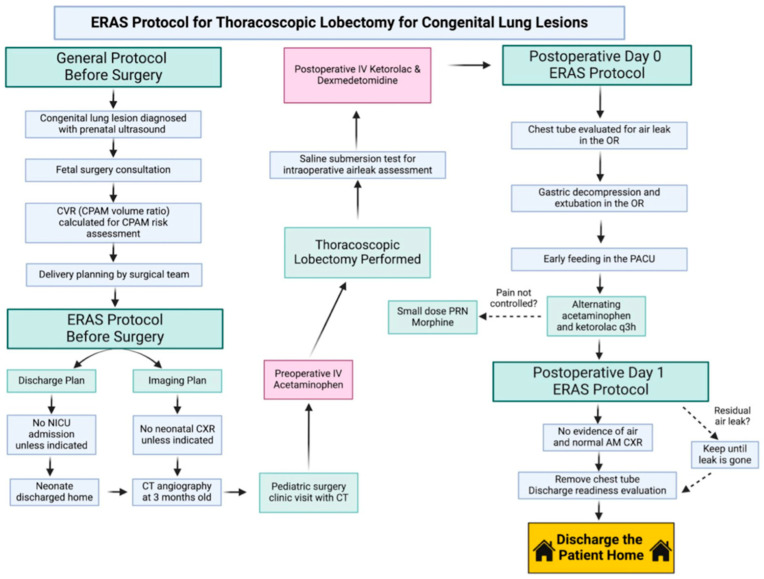
Step-by-step breakdown of the Enhanced Recovery After Surgery (ERAS) protocol for pediatric thoracoscopic lobectomy. The protocol includes standardized components across the preoperative, intraoperative, and postoperative phases of care, emphasizing multimodal analgesia, early mobilization, optimized fluid management, and coordinated discharge planning.

Figure 1 depicts the detailed components of our ERAS protocol. Briefly, our protocol consisted of a standardized approach for perinatal counseling of families, a standardized pre-operative approach to surgical planning, intraoperative measures to ensure there were no air leaks or airway mucus plugs, and finally a standardized post-operative approach to pain control and timing of chest tube removal based on clinical signs. Statistical analysis on all continuous variables was assessed using an independent samples *t*-test, and all categorical variables were analyzed using a chi-square test of independence using SPSS (IBM Statistical Software v.30; Chicago, IL, USA). Two patients in the non-ERAS cohort were excluded from LOS analysis as their prolonged stay was due to medical and social events unrelated to surgery. All effect sizes are reported as Cohen’s d for continuous variables, and Phi is reported for categorical variables.

## 3. Results

**Figure 2:**
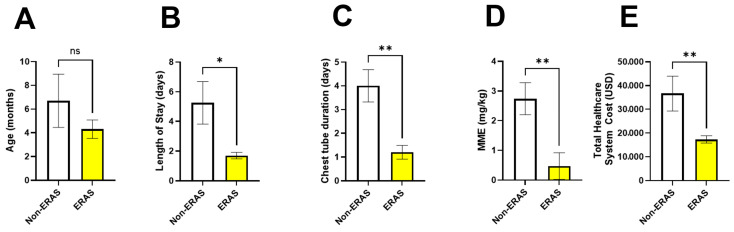
Comparison of perioperative outcomes between patients managed with the ERAS protocol (yellow) and those managed without ERAS (white). (**A**) Patient age at the time of surgery showed no significant difference between groups. (**B**) Length of hospital stay was also significantly reduced in the ERAS group (* *p* < 0.05). (**C**) Chest tube duration was significantly reduced in the ERAS group (** *p* < 0.01). (**D**) Total morphine mg equivalent (MME) throughout the hospital stay was also significantly reduced in the ERAS group (** *p* < 0.01). (**E**) Use of the ERAS protocol leads to decreased cost to healthcare system in United States Dollars (USD; ** *p* < 0.01). Data are presented as mean ± standard deviation. Statistical significance was determined by an independent samples *t*-test. The Non-ERAS cohort consisted of N = 10 patients, and the ERAS cohort consisted of N = 10 patients.

There were no significant differences in relevant variables between open and thoracoscopic patients in the non-ERAS cohort (Supplemental Figure S1A–E), hence these patients were combined into a common non-ERAS group. Patients included within the ERAS protocol tended to be younger at the time of surgery, with ERAS patients receiving surgery at an average of 4.25 ± 2.76 months of age, whereas the non-ERAS patients received surgery at an average of 6.45 ± 6.78 months of age (Figure 2A, *p* = 0.40, Cohen’s d = 0.44). Importantly, patients in the ERAS cohort were sent home 3.5 days earlier, with an average length of stay of 1.77 ± 0.60 days post-operation compared to the non-ERAS cohort whose hospital stay was an average of 5.25 ± 3.79 days (Figure 2B, *p* = 0.03, Cohen’s d = 1.9). Moreover, patients in the ERAS protocol had significantly shorter chest tube durations with an average of 1.44 ± 0.73 days compared to the average chest tube duration of the non-ERAS group, 3.64 ± 2.38 days (Figure 2C, *p* = 0.02, Cohen’s d = 1.4, independent samples *t*-test). There was no difference in the morphine milligram equivalent (MME, *p* = 0.36) or total cost to the healthcare system (*p* = 0.39) between the non-ERAS group who received thoracoscopic versus thoracotomy procedures. Therefore, we combined these into a single non-ERAS group for comparison of MME and healthcare system costs. Patients within the ERAS protocol received a lower total MME throughout their entire hospital stay, receiving 0.47 ± 1.71 mg/kg, compared to non-ERAS patients who received an average of 2.74 ± 1.41 mg/kg (Figure 2D, *p* = 0.0046, Cohen’s d =1.45). Use of this ERAS protocol shows promise of also reducing the overall cost to healthcare systems by about 50%, with the average cost to our hospital system being $17,462 ± $5174 in the ERAS group, whereas the non-ERAS group cost the hospital system $33,210 ± $16,923 (Figure 2E, *p* = 0.0037, Cohen’s d = 1.26).

**Figure 3:**
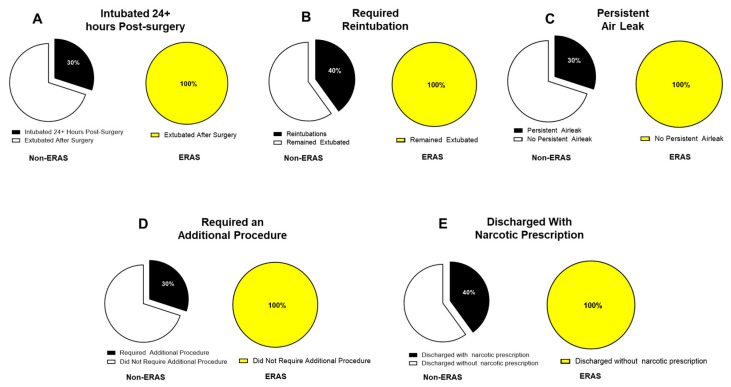
Postoperative complication rates in ERAS versus Non-ERAS patients. (**A**) Percentage of patients remaining intubated more than 24 h after surgery. (**B**) Percentage of patients requiring reintubation. (**C**) Percentage of patients that suffered a persistent air leak (>48 h). (**D**) Incidence of additional procedures required postoperatively. (**E**) Percentage of patients discharged with a narcotic prescription. In all categories shown, no ERAS patients experienced these complications, while notable proportions were observed in the non-ERAS group. Data are presented as percentages. Statistical significance was determined by a chi-squared test of independence. The Non-ERAS cohort consisted of N = 10 patients, and the ERAS cohort consisted of N = 10 patients.

All patients in the ERAS protocol were successfully extubated in the operating room directly after their surgery, whereas 3 patients in the non-ERAS protocol were extubated at least 24 h post-operation (Figure 3A, *p* = 0.06, Phi = 0.42). None of the ERAS patients required re-intubation for any reason, whereas 4/10 patients in the non-ERAS group required re-intubation at some point before their discharge (Figure 3B, *p* = 0.03, Phi = 0.51). None of the ERAS cohort had a persistent air leak (defined as an air leak lasting 2 or more days post-op), whereas 3/10 non-ERAS patients had a documented persistent air leak (Figure 3C, *p* = 0.06, Phi = 0.42). None of the patients in the ERAS protocol required an additional procedure during their initial hospital stay, whereas 3/10 separate non-ERAS patients required an additional procedure: 2 bronchoscopic mucous plug removals and 1 reoperation to complete the lobectomy following subsegmental resection (Figure 3D, *p* = 0.06, Phi = 0.42). None of the ERAS patients were discharged with a narcotic prescription, whereas 4/10 patients in the non-ERAS group (2 thoracotomy, 2 thoracoscopic) were sent with an oxycodone prescription (Figure 3E, *p* = 0.03, Phi = 0.51). Overall, none of the ERAS patients had any Clavien–Dindo complications, whereas 5/10 patients in the non-ERAS group had at least one Clavien–Dindo complication, with each patient’s complication summarized in Table 2 (*p* = 0.01, Phi = 0.58).

Table 2 Postoperative complications classified by Clavien–Dindo grade. All complications occurred in the non-ERAS group, including respiratory and thoracic complications requiring reintervention. No ERAS patients experienced Clavien–Dindo grade complications.

Participants were analyzed according to national and state reports on social determinants of health (SDoH) by their respective addresses. We found that overall, 11 out of 20 patients were categorized as very low to low on the State and National Childhood Opportunity Index (COI). Additionally, 14 had a social vulnerability index (SVI) of low-medium to medium-high. The mean state Area Deprivation Index (ADI) of the cohort was 6 (on a scale of 1–10) and nationally found to be 76 (on a scale of 1–100). Specifically, 15 participants had an ADI score of 60 or greater, and 7 of those had a “high” ADI score of 80 or above, indicating significant levels of socioeconomic deprivation within both cohorts of patients. When stratified by whether or not the participant underwent ERAS protocol, we found that those in the non-ERAS cohort, on average, lived in communities with lower state and national COI and higher SVI compared to the ERAS cohort. ERAS cohort revealed a slightly higher national ADI mean at 76 compared to the non-ERAS at 70. The state ADI mean was 6 in each group. After excluding two patients who traveled from out-of-state, ERAS patients typically lived closer to the hospital, with an average one-way travel distance of 36 ± 47 miles, while non-ERAS patients had a one-way travel distance of 68 ± 58 miles (*p* = 0.21). Travel distance did not influence discharge timing. Discharge was determined by clinical readiness, and one ERAS patient discharged on postoperative day 1 resided farther from the hospital than most pre-ERAS patients (7 of 10), indicating that distance was not a limiting factor.

## 4. Discussion

Our results demonstrate the successful design and implementation of an ERAS protocol for pediatric patients undergoing thoracoscopic resections for congenital cystic lung lesions. Implementing the ERAS protocol was safe, resulting in a significant decrease in hospital length of stay (LOS) and chest tube duration without an increase in complication rate. These efforts began after observing heterogeneous management strategies and outcomes in pediatric lung lesion resections at the University of Missouri, prior to the implementation of these protocols. The baseline rate of symptomatic patients did not differ significantly between cohorts, supporting that the observed reductions were associated with the ERAS pathway rather than underlying differences in presentation. Our protocol began with prenatal counseling of patients seen at our MU Health Care Fetal Intervention Center. Upon delivery, patients diagnosed with a congenital lung lesion were not admitted to the neonatal intensive care unit unless clinically indicated.

All patients in the ERAS protocol received CT angiography at 3 months of age, with surgery scheduled a few weeks to a month following the CT angiography, whereas in our non-ERAS group, there was greater variation in the timing of CT angiography and surgical resection. Patients with very small lesions on the CT were offered follow-up rather than resection. Although mixed results have been reported, some previous studies suggest that there may be a slight advantage to earlier resection, particularly in terms of long-term pulmonary function, due to the pediatric lung’s impressive ability to undergo post-lobectomy compensatory lung growth [13,14,15,16,17]. Moreover, with there being evidence of lower morbidity and shorter operative times in patients who receive earlier lobectomy [13,14,15], our approach for this ERAS protocol was to opt for an earlier procedure at 3–4 months of age. Indeed, the American College of Surgeons recommends that all pediatric surgical specialties adopt standardized protocols for preoperative evaluation and management to achieve more predictable outcomes, improved efficiency, and fewer complications [16].

In addition to a standardized surgical planning approach, perhaps more crucial to an ERAS protocol is standardizing the surgical approach itself; in this case, a lobectomy for resection of a congenital lung lesion early in life, following American Pediatric Surgical Association guidelines [3]. Marinucci and colleagues have reported that lobectomies for the treatment of CPAMs have been shown to have lower CPAM recurrence rates, as well as lower complication rates [17]. Indeed, one of our patients in the non-ERAS group received a subsegmental resection, which led to a late air leak and eventual tension pneumothorax requiring reintervention with completion lobectomy.

Another key element of our protocol is intraoperative air leak assessment with 40 cm H_2_O pressure after limited endotracheal tube lavage to rule out any mucus/blood clot clogging. Lack of intraoperative air leak allows for expedited chest tube removal, resulting in earlier hospital discharge and changes the dogma of water sealing the chest tube followed by repeat chest *x*-rays. In support of this practice, several studies have found that chest tube reinsertion is uncommon and is often driven by symptomology rather than routine radiographic findings [18,19,20,21]. Thus, our ERAS protocol also safely decreases post-operative chest *x*-rays—limiting exposure to radiation in children.

In congruence with the general recommendation of ERAS protocols to utilize multimodal pain control, we chose to adopt a similar analgesic protocol recommended by the American Association of Thoracic Surgery, which utilized intravenous acetaminophen, ketorolac, and dexmedetomidine for pediatric cardiac ERAS protocols [10]. On the day of the procedure, all patients received preoperative IV acetaminophen (15 mg/kg) and postoperative ketorolac (0.5 mg/kg) and dexmedetomidine (0.25–1 µgram/kg) followed by scheduled alternating acetaminophen (15 mg/kg) and ketorolac (0.5 mg/kg) postoperatively. These analgesic strategies have also been shown to reduce opioid consumption and reduce the length of stay in other ERAS protocols, which was one of our main goals with the implementation of this protocol [11,12,22,23,24,25,26]. This is especially crucial in infants to allow another key element of ERAS protocols, early nutritional support. All patients had gastric decompression intraoperatively to counteract the incidence of acute gastric distention post lobectomy. The quality pain control efforts, along with streamlined and standardized extubation in the operating room, allowed babies in our ERAS cohort to be fed by our care team and their parents in the PACU almost immediately after being weaned from anesthesia.

Overall, by implementing this ERAS protocol at our institution, we were able to provide a quality, standardized surgical approach for patients with congenital lung malformations. Compared to the non-ERAS group, patients who received thoracoscopic pulmonary resections for congenital cystic lung lesions after implementation of the ERAS protocol had significantly quicker removal of their chest tube (2 days earlier) and expedited hospital discharge (3.5 days earlier). Most importantly, we attained these improved results without an increased rate of complications despite a younger age at surgery in the ERAS cohort.

The literature reports a wide range for the average post-operative length of stay for CPAM resection [27,28,29,30,31,32]. In contrast to the most recent large-scale analysis from Raymond et al. who have shown an average hospital LOS of 3 days for thoracoscopic lobectomies for CPAM removal [33], our ERAS patients were discharged on average 1.7 days post operatively. Some studies have reported successful same-day discharge for non-lobectomy cases [34].

The implementation of Enhanced Recovery After Surgery (ERAS) protocols has been shown to improve patient outcomes across various surgical disciplines. However, their adoption is often met with institutional and logistical challenges, including variability in provider practices, resistance to change, and difficulties in standardizing care across multidisciplinary teams. At our institution, we recognized these hurdles early in the development process and sought to address them through proactive coordination, data-driven equipoise, stakeholder inclusion, and development of evidence-based standardized clinical practice protocols.

General practices for the care of congenital lung lesions have evolved from open procedures (thoracotomy) to most resections now being performed thoracoscopically [4]. We observed a similar transition in our pre-ERAS patients, with thoracoscopic procedures becoming the preferred surgical approach at our institution. However, this did not dramatically change the outcomes tracked in this study (see Supplementary Figure S1A–E), which led to our implementation of an ERAS protocol in this patient population. The education and implementation period of the protocol involved floor nurses receiving just-in-time educational presentations delivered by nurse practitioners on the pediatric surgical team, with surgical resident education being managed by the attending surgeons overseeing the protocol using the same presentation and walk-through. One of the key barriers we encountered early in the implementation process was inconsistency in perioperative management, resulting from variability in clinical workflows and provider preferences. To mitigate this, we developed a standardized order set within the electronic medical record (EMR) system for patients undergoing thoracoscopic lobectomy. This allowed all members of the care team, such as the surgeons, anesthesiologists, nurses, and pharmacists, to access and implement the appropriate medications, dosages, and timing parameters with consistency. By creating a unified care pathway, we were able to enhance communication across disciplines and ensure the timely implementation of protocol components, including pain management, fluid resuscitation, and early mobilization. However, there was still one notable lapse in our ERAS protocol, with one patient receiving a total of 4.5 MME/kg throughout their hospital stay on parental request due to concerns about pain. Compliance is a persistently recognized problem across ERAS protocols, with postoperative elements such as mobilization, nutrition, catheter/drainage removal, and minimization of pain medications, among others, representing the most common areas of protocol lapse [35,36,37,38,39,40]. Parent and caregiver preferences may be an additional implementation barrier specifically observed in pediatric ERAS protocols and likely warrant further investigation.

We observed some variability in the timing of postoperative milestones among patients, which can be attributed to individual patient factors and the learning curve associated with the early stages of protocol adoption. Indeed, as the protocol matured and the comfort level grew, we began to consistently send patients home on postoperative day 1 rather than postoperative day 2—with the last 4 consecutive patients being discharged on postoperative day 1. We intend to continue refining our ERAS protocol and prospectively evaluating its outcomes. Indeed, we have already begun assessing the feasibility of a prospective no-pleural-drain approach, which will allow patient discharge on postoperative day 1. This poses the critical question and dilemma of whether a thoracoscopic lobectomy in pediatric patients can transition towards becoming a same-day surgery in select patients. In addition to the continued refinement of our ERAS protocol, future studies will analyze the effect of our ERAS protocol on the overall financial and healthcare burden for both our patients and the hospital system. Patients not included in the ERAS protocol tended to have lower social determinants of health, in addition to traveling longer distances and experiencing longer hospital stays. These findings highlight potential socioeconomic barriers to engagement with perioperative care pathways. While ERAS protocols may improve efficiency and recovery, implementation must be individualized, taking into account the patient’s clinical status, caregiver support, and home resources to ensure safe early discharge for all patients.

### Limitations

The limitations of our study include a small sample size and the retrospective, single-center design, which may affect the generalizability of our findings. Our sample size was insufficient to support a stable multivariable regression model, particularly given that the non-ERAS cohort was further divided into thoracoscopic and thoracotomy subgroups. As a result, we used a one-way ANOVA to compare group means, which we felt was the most appropriate statistical approach for a dataset of this size and provided rationale for combining the two approaches into a single non-ERAS group given the similarity in outcomes measured herein. Some prenatal variables, such as complete CVR measurements for several patients in the non-ERAS cohort, were unavailable due to limited access to records from outside referring hospitals. The non-ERAS and ERAS cohorts were separated chronologically, with ERAS patients treated after 2022, which introduces potential temporal bias related to learning curves, increasing team experience, and evolving anesthesia practices. Importantly, aside from the differences in surgical approach and the ERAS streamlining protocol, all other aspects of perioperative care were consistent between the cohorts. Additionally, long-term outcomes and cost-effectiveness metrics were not included in this initial analysis and should be considered in future studies. A prospective institutional study is currently underway, which will enable the generalizability of these findings, quantify compliance rates in larger cohorts, and identify any additional barriers to protocol adherence.

## 5. Conclusions

In summary, we report the successful development and integration of an ERAS protocol for pediatric thoracoscopic pulmonary resections at our institution. The implementation of this protocol resulted in improved perioperative outcomes compared to either open or thoracoscopic cases performed prior to implementation of the ERAS protocol, including earlier chest tube removal, reduced hospital length of stay, decreased opioid use, and a lower overall healthcare burden for both patients and the hospital system. Our experience demonstrates that common barriers to ERAS implementation can be effectively overcome.

## Figures and Tables

**Table 1:** Patient Characteristics.

Characteristic	ERAS (n = 10)	Non-ERAS (n = 10)	*p*-Value
**Female**	3 (30%)	4 (40%)	*p* = 0.99
**Age (months)**	4.25 ± 2.76	6.45 ± 6.78	*p* = 0.41
**Race**			*p* = 0.53
White	9	8	
Black	1	2	
**Hybrid Lesion**	4 (40%)	2 (20%)	*p* = 0.63
**Approach**			*p* = 0.03
Thoracoscopic	10	5	
Thoracotomy	0	5	
**Lesion Type**			*p* = 0.05
Type 1	1 (10%)	6 (60%)	
Type 2	8 (80%)	3 (30%)	
CLE	-	1 (10%)	
eBPS *	1 (10)	-	
**Lobe/Sidedness**			*p* = 0.31
RUL	1 (10%)	2 (20%)	
RML	-	1 (10)	
RLL	2 (20%)	2 (20%)	
LUL	-	2 (20%)	
LLL	7 (70%)	3 (30%)	

Patient demographics for ERAS and non-ERAS patients undergoing lobectomy procedures under the ERAS protocol. CLE: Congenital Lumbar Emphysema, eBPS: Extralobar Bronchopulmonary Sequestration. * Indicates that the eBPS patient did not receive a lobectomy, but a thoracoscopic resection and arterial ligation.

**Table 2:** Clavien–Dindo Complication Classification.

Clavien–Dindo Class	Complication Description
3b	Severe mucous plug requiring bronchoscopic removal
4a	Reintubated due to poor chest expansion
4a	Reintubated due to respiratory distress
3a	Pneumothorax requiring chest tube placement
3b	Return to OR due to tension pneumothorax

5/10 Non-ERAS Patients had Clavien–Dindo complications. 0/10 ERAS patients had Clavien–Dindo complications.

## Data Availability

The data presented in this study are available on request from the corresponding author. Data in this study are part of an ongoing prospective study as comparison.

## Supplementary Materials

The following supporting information can be downloaded at: https://www.mdpi.com/article/10.3390/children12121658/s1, Figure S1: Comparison of perioperative outcomes between patients managed without ERAS protocol receiving thoracotomies (Non-ERAS TC; white) without ERAS protocol receiving thoracoscopic surgeries (Non-ERAS TS; grey), and those managed with the ERAS protocol (yellow). (A) Patient age at the time of surgery showed no significant (ns) difference between groups. (B) Length of hospital stay did not differ between the two non-ERAS groups or the ERAS group. (C) Chest tube duration was significantly reduced in the ERAS group (* *p* < 0.05) compared to the two non-ERAS groups. (D) There was no significant difference between the two non-ERAS groups chest tube duration. (E) There was no significant difference between the two non-ERAS groups total cost to healthcare system in United States Dollars (USD). Data are presented as mean ± standard deviation. Statistical significance was determined using a One-Way ANOVA. The Non-ERAS TC cohort consisted of N = 5 patients, the Non-ERAS TS cohort consisted of N = 5 patients and the ERAS cohort consisted of N = 10 patients.

## Abbreviations

The following abbreviations are used in this manuscript:

ERAS	Enhanced Recovery After Surgery
CPAM	Congenital Pulmonary Airway Malformation
LOS	Length of Stay
SDoH	Social Determinants of Health
