# Supplementary figures and images for: Enhanced Recovery After Surgery for Pediatric Lung Resection: Effects of a New Protocol
Source: Children (Basel). 2025 Dec 7;12(12):1658. doi: 10.3390/children12121658 (PMC12732230; doi:10.3390/children12121658)

**A**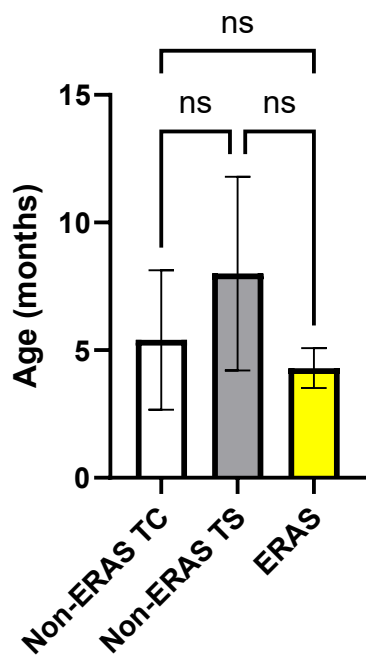**B**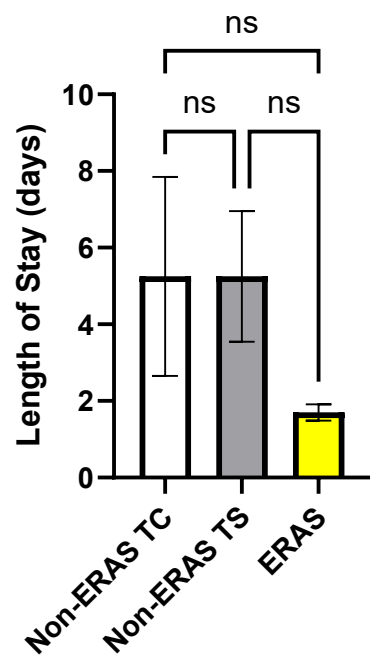**C**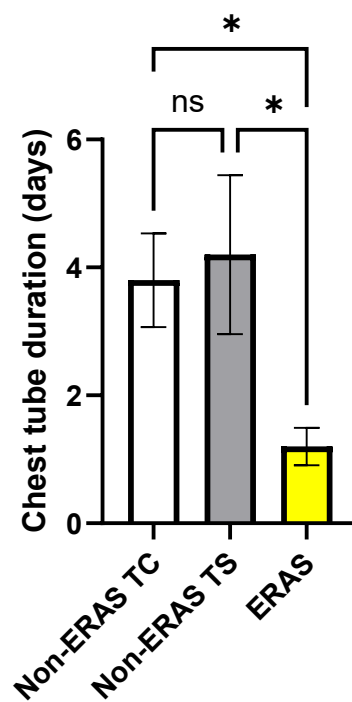**D**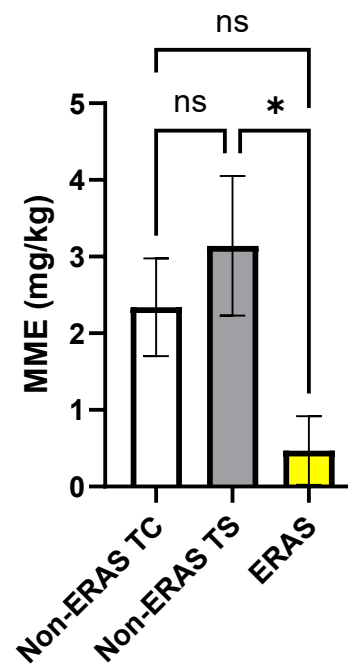**E**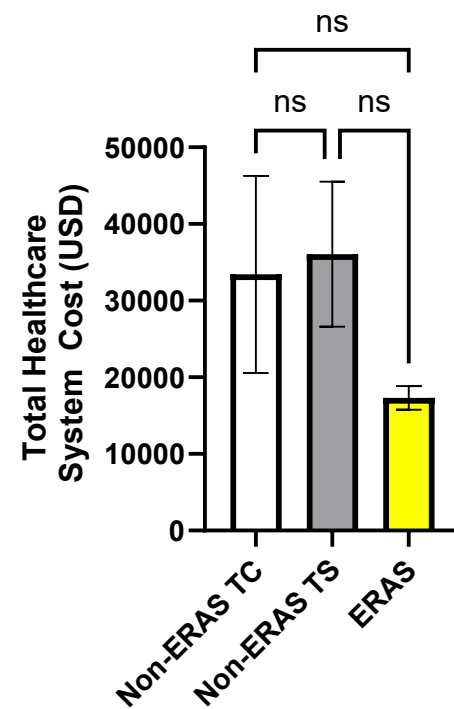

Supplement: Supplementary file 1 [file children-12-01658-s001.zip › children-3970201-supplementary.pdf]
